# Divergent structural and functional brain alterations in HIV-infected patients: a multimodal meta-analysis

**DOI:** 10.3389/fneur.2025.1618408

**Published:** 2025-08-18

**Authors:** Zhong Li, Xingxing Jin, Meng Zhang, Hongxia Wang, Wangyi Liu, Beiran Wang, Baolin Wu, Xuekun Li

**Affiliations:** ^1^Department of Magnetic Resonance, The First Affiliated Hospital of Xinxiang Medical University, Xinxiang, China; ^2^Department of Radiology, West China Hospital, Sichuan University, Chengdu, China

**Keywords:** human immunodeficiency virus, gray matter volume, resting state, brain activity, meta-analysis

## Abstract

Neuroimaging studies have identified brain structural and functional alterations in HIV-infected patients; however, the results are inconsistent. This study aimed to characterize the effects of HIV infection on regional gray matter volume (GMV) and resting-state brain activity, and to further investigate the relations between abnormalities in these two modalities. We conducted voxel-wise meta-analysis of voxel-based morphometry (VBM) and functional studies, respectively, to identify regional GMV and brain activity alterations in HIV-infected patients. Multimodal analysis was performed to examine the overlap of regional GMV and brain activity alterations. Meta-regression analysis was conducted to evaluate the potential effects of clinical variables. Eleven whole-brain VBM studies and eight resting-state functional studies were included. HIV-infected patients showed structural abnormalities alone in the bilateral medial prefrontal cortex/anterior cingulate cortex, bilateral calcarine cortex and left amygdala, and had functional abnormalities alone in the left middle frontal gyrus, left parahippocampal gyrus, left superior temporal gyrus and visual cortices. No conjoint brain structural and functional abnormalities were identified. This study characterized dissociated brain structural and functional alterations in HIV-infected patients from a perspective of multimodal meta-analysis, which may provide new insights into the neurobiology of HIV-associated neurocognitive impairment.

## Introduction

Human immunodeficiency virus (HIV) and acquired immune deficiency syndrome (AIDS) cause substantial disease burden and mortality (1). As a neurotropic virus, HIV can infiltrate the central nervous system at early stage of infection, which is often associated with various neurological complications, such as HIV-associated neurocognitive disorders (HAND) (2). Although antiretroviral therapy (ART) has gradually made AIDS a manageable chronic disease and greatly decreased mortality due to HIV infection, HAND remains prevalent in antiretroviral-treated people living with HIV (3). Such neurocognitive deficits reduce quality of life and present a significant challenge to clinicians in the context of an ageing HIV population with a growing number of comorbidities (4). The key features of HAND in HIV infection are cognitive impairments, which include notable deficits in attention, working memory, motor function, and visual perception (5). Unfortunately, the neural mechanisms underlying these deficits has not been fully characterized. To better monitor diagnosis and disease progression, and to comprehensively understand the disease, it is essential for us to identify the neural bases of cognitive deficits in HIV infection.

Magnetic resonance imaging (MRI) has provided a valuable and noninvasive tool to investigate brain morphometric and functional alterations in HIV infection. Structural MR imaging technique can be used to detect anatomical alterations in HIV infection. Recent review of structural MRI studies has summarized that HIV infection is associated with atrophy in the subcortical and limbic regions, such as caudate nucleus, putamen, and hippocampus (6). Quantitative meta-analysis also revealed neurostructural changes related to serostatus in HIV-infected participants, with total brain volume, total gray matter volume, and cerebrospinal fluid volume showing reliable serostatus effects (7). Notably, HIV-associated structural abnormalities, especially gray matter deficits in the subcortical and limbic structures are based on a region-of-interest (ROI) method. An important technical issue is that structural neuroimaging studies based on a ROI strategy are inherently biased; by contrast, voxel-based morphometry (VBM), a whole-brain, unbiased technique for analyzing structural MR images, can characterize regional cerebral volume and tissue concentration differences at a voxel level. Meanwhile, resting-state functional MRI (rs-fMRI) technique is a promising technique to detect local features of the spontaneous blood oxygenation level-dependent signal. Amplitude of low-frequency fluctuation (ALFF) and regional homogeneity (ReHo) are both widely used and complementary metrics for assessing regional brain activity in rs-fMRI studies. Theoretically, ALFF quantifies the intensity of low-frequency oscillations in spontaneous neural activity, while ReHo reflects the statistical similarity of spontaneous neural activity among spatially adjacent brain tissues.

Based on whole-brain level voxel-based analyses, abnormal GMV and intrinsic neural activity have been detected in persons living with HIV infection, even in the era of combination ART. However, the volumetric and resting-state brain activity alterations detected in HIV-infected patients have been inconsistent and are poorly replicated for some brain regions. For example, using whole-brain voxel-based morphometry (VBM) analysis, some structural MRI studies found regional GMV reductions in the cortical structures, including the anterior cingulate cortex (ACC) (8–10), orbitofrontal cortex (9, 11), and dorsolateral prefrontal cortex (PFC) (12) in HIV-infected patients; while other studies of HIV infection reported GMV deficits in the subcortical regions, such as hippocampus and thalamus (13, 14). Reduced GMV in the cerebellum (10, 15), which is involved in cognitive processing (16), has also been reported in HIV-infected patients. Additionally, rs-fMRI studies of HIV infection have reported abnormal brain activity in the cortical and subcortical regions. However, findings from these studies have been less consistent than expected, and some results are even the opposite. For example, brain activity alterations in the sensorimotor cortex (e.g., precentral/postcentral gyrus) in HIV-infected patients relative to controls have been controversial. Decreased brain activity (17–19), increased brain activity (20), or null findings (21) in these regions have been reported. These inconsistent findings highlight the need to characterize the replicable and reliable brain structural and functional abnormalities.

Neuroimaging meta-analysis is a powerful method for summarizing and integrating findings across various studies. Anisotropic effect size-signed differential mapping (AES-SDM), a quantitative coordinate-based neuroimaging meta-analytic approach, has been widely used to identify consistent structural and functional brain abnormalities (22–25). AES-SDM has the advantages of providing more precise spatial localization, integrating both positive and negative effects within the same analysis, accounting for spatial heterogeneity through anisotropic kernels, and enhancing the sensitivity and specificity of meta-analytic findings in neuroimaging studies (26).

Thus, we aimed to conduct separate voxel-based meta-analyses of VBM and rs-fMRI studies to identify the most consistent and replicable regions with abnormal GMV and brain activity in HIV infection using AES-SDM. Furthermore, we aimed to conduct a multimodal meta-analysis of VBM and rs-fMRI studies to determine whether HIV-infected patients exhibit brain regions with both structural and functional abnormalities. Finally, exploratory meta-regression analyses were performed to evaluate the potential effects of demographic and clinical variables on identified brain structural and functional alterations.

## Methods

### Selection of eligible studies

We performed the meta-analysis based on the Preferred Reporting Items for Systematic Reviews and Meta-Analysis (PRISMA) guidelines (Supplementary Table S1). A comprehensive literature search strategy was applied to select pertinent studies up to December 2024 in PubMed, Web of Science and EMBASE databases. The keywords (“human immunodeficiency virus” or “HIV”) plus (“gray matter” or “grey matter” or “GM” or “voxel-based morphometry” or “VBM” or “voxel-wise” or “voxel-based” or “volumetric” or “morphometry”) and (“magnetic resonance imaging” or “MRI” or “MR imaging” or “neuroimaging”) were used to identify candidate VBM studies. rs-fMRI studies were identified using the keywords (“human immunodeficiency virus” or “HIV”) plus (“amplitude of low-frequency fluctuation” or “regional homogeneity” or “fractional amplitude of low-frequency fluctuation” or “ALFF” or “ReHo” or “fALFF”) and (“magnetic resonance imaging” or “MRI” or “MR imaging” or “functional MRI” or “fMRI” or “neuroimaging”). Furthermore, the reference lists of the retrieved eligible articles and review articles were also searched manually to acquire additional relevant articles for inclusion.

A study was included if it: (1) was published as an original article in a peer-reviewed English language journal; (2) used a whole-brain level voxel-based analysis to detect GMV, ALFF, fALFF, or ReHo changes between HIV-infected patients and HIV-negative controls; and (3) clearly reported whole-brain three-dimensional Montreal Neurological Institute (MNI) or Talairach coordinates (*x, y, z*) of the altered brain regions. We excluded studies that was published as a case report, letter, abstract, review, or meta-analysis. We also excluded studies that reported only specific region-of-interest (ROI) findings, studies that not compared with a HIV-negative control group and studies from which peak coordinates could not be retrieved even after contacting the authors by email or telephone. For studies containing multiple independent patient samples, the appropriate coordinates were included as separate studies. Additionally, we only included the baseline data in the longitudinal studies. For studies using overlapping samples, the study with the largest sample size was included. Two authors (B. L. W. and Z. L.) independently performed the literature searches, selected the articles that meet the inclusion criteria. Afterwards, the results were compared, and for any articles with inconsistencies, the authors consulted to reach an agreement.

### Data extraction

For each included study, the demographic and clinical characteristics (sample size, age, gender, and current CD4 cell count) and technique details (analytic method, magnetic field strength, software for data analyses, smoothing kernel, statistical threshold, and coordinate system) were recorded. Data extraction was independently performed by two authors (B. L. W. and Z. L.), and any disagreement was resolved by discussion.

### Quality assessment

The qualities of the included studies were evaluated using a 10-point checklist (Supplementary Table S2). This checklist was divided into three categories: (1) the quality of the detailed information of participants, including the quality of the specific diagnostic criteria, the demographic and clinical variables (e.g., age, gender, illness duration), and the sample size; (2) the quality of methods for image acquisition and analysis; and (3) the quality of results and conclusions. The quality assessment of each included study was independently evaluated by two authors (B. L. W. and Z. L.), and any disagreement was resolved by discussion.

### Meta-analyses of structural and functional alterations

Two independent voxel-wise meta-analyses of the included structural and functional studies were conducted to separately identify the differences in GMV and resting-state brain activity between HIV-infected patients and HIV-negative controls using the AES-SDM software.^1^ The AES-SDM can combine the reported peak coordinates extracted from the included studies with statistical parametric maps using effect sizes, and then it recreates original maps of the effect size of neural activity differences between patients and controls, the details and instructions of this meta-analytic method has been described in the AES-SDM tutorial.^2^ Therefore, we summarized a brief description here. First, the reported peak coordinates and their corresponding effect sizes (derived, e.g., from *t* statistics) were extracted from each included study, and then created an SDM table to collect demographic data and clinical variables. Then, the original effect-size brain maps of each included study were recreated to generate voxel-level Monte Carlo brain maps. Finally, a mean map was obtained using a voxel-wise calculation in a random-effects model, weighted by sample size and intra-study variance, and the inter-study heterogeneity was also taken into consideration. To obtain more stable and reliable results from the meta-analysis, we set the permutation at 20 and applied a standard SDM threshold (voxel-wise *p* < 0.005 with SDM-Z > 1, and cluster size > 20 voxels). Multimodal analysis was performed to identify the overlapped regions with both structural and functional abnormalities in HIV-infected patients (Supplementary materials).

### Reliability analysis

To test the reliability of our findings, systematic whole-brain voxel-based jackknife sensitivity analyses were conducted in each independent meta-analysis. This process was achieved by iteratively repeating the same statistical analysis *n* (*n* = datasets) times, discarding a different dataset each time (26, 27). A brain region is regarded as highly replicable if it remains significant in all or most of the combinations of the datasets.

### Heterogeneity and publication bias analyses

We analyzed the between-study variations to assess the heterogeneity of the identified WM abnormalities using a random-effects model with *Q* statistics and tested with a permutation approach (voxel-wise threshold *p* = 0.005, SDM-Z = 1, cluster extent threshold = 20 voxels). For each significant cluster, we used Egger tests to assess the asymmetry of funnel plots to examine publication bias (*p* < 0.05 indicated publication bias).

### Meta-regression analysis

The potential effects of relevant demographic and clinical variables (mean age, percentage of males and mean current CD4 cell count) on regional brain structural and functional alterations in HIV-infected patients by a random-effects general linear meta-regression. In order to minimize the possibility of detecting spurious associations, a stringent threshold of *p* < 0.0005 was used to determine statistical significance. The clusters showing significant alterations were reported only when they were found in both the slope and one of the extremes of the regressor, and the regions that were not detected in the main analysis were discarded (27, 28).

## Results

### Included studies and sample characteristics

**Figure 1:**
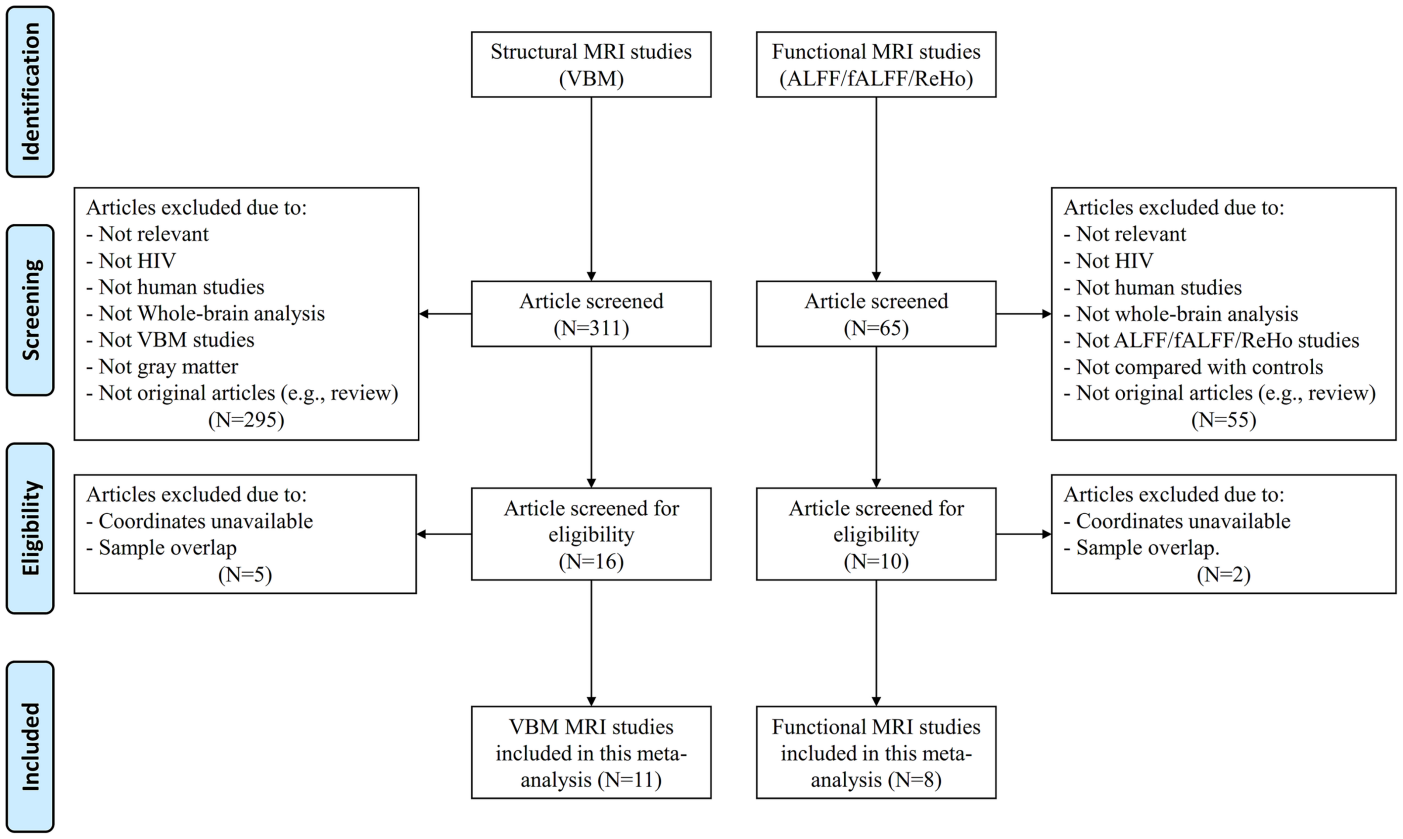
Flow diagram of study selection, based on PRISMA guidelines. VBM, Voxel-based morphometry; ALFF, Amplitude of low-frequency fluctuation; fALFF, Fractional amplitude of low-frequency fluctuation; ReHo, Regional homogeneity.

The detailed flow chart of studies inclusion is shown in Figure 1. Twelve whole-brain VBM studies (8–15, 29–32) and eight rs-fMRI studies (17–21, 32–34) met the inclusion criteria. One VBM study was discarded due to sample overlap (31). Finally, 11 VBM studies comprising 459 HIV-infected patients (383 males and 76 females) and 385 HIV-negative controls (302 males and 83 females), and eight rs-fMRI studies comprising 229 HIV-infected patients (202 males and 27 females) and 235 HIV-negative controls (204 males and 31 females) were included in the current meta-analysis.

The detailed demographic data and clinical characteristics are shown in Table 1. None of the included VBM or rs-fMRI studies reported significant differences in age and gender ratio between HIV-infected patients and HIV-negative controls. The technique details of included VBM and rs-fMRI studies are summarized in Supplementary Tables S3, S4.

**Table 1:** Demographic and clinical characteristics of subjects of studies included in the meta-analysis.

Studies	HIV-infected patients					Controls		Measure	Quality score (out of 10)
	*N*	%Male	Age, year	Current CD4+ cell count, cells/ml	Medication, %	*N*	Age, year		
Voxel-based morphometry studies									
Küper et al. (8)	28	85.7	51.2	555	ART, 94	48	48.2	GMV	9.5
Li et al. (9)	36	100	34.5	206	ART, 30.6	33	31.4	GMV	9.5
Wilson et al. (29)	17	76.5	56.8	748	ART, 100	17	57.8	GMV	9
Wang et al. (11)	26	88.5	38.0	255	ART, 34.6	26	34.0	GMV	9
Zhou et al. (12)	22	95.5	38.2	151	Unmedicated	22	34.7	GMV	9.5
Sanford et al. (30)	125	64.0	47.2	533	ART, 90	62	45.4	GMV	10
Li et al. (10)	24	58.3	15.0	598	ART, 100	33	14.8	GMV	9.5
Yu et al. (15)	16	50	13.6	559	ART, 100	25	13.3	GMV	9
Liu et al. (13)	91	98.9	31.5	483	NA	46	34.4	GMV	9.5
Kato et al. (14)	31	100	42.6	574	ART, 100	33	41.1	GMV	9
Ma et al. (32)	43	100	28.2	480	ART, NA	40	27.8	GMV	9.5
Functional studies									
Wang et al. (20)	13	61.5	15.1	642	ART, 100	22	15.4	ReHo	9
Yadav et al. (17)	26	53.8	9.9	490	ART, 100	20	8.8	ALFF	9
Bak et al. (18)	12	100	52.5	704	ART, 100	11	53.0	ALFF	9
Egbert et al. (33)	54	100	41.4	574	ART, 100	54	42.8	ReHo	9.5
Li et al. (19)	26	92.3	33.1	524.8	ART, 100	25	33.2	ALFF	9.5
Sarma et al. (21)	11	27.3	22.5	507	ART, 100	16	22.5	ALFF	9
Ma et al. (32)	43	100	28.2	480	ART, NA	40	27.8	ALFF	10
Han et al. (34)	44	100	30.2	516	NA	47	31.0	ReHo	10

ART, Antiretroviral therapy; GMV, Gray matter volume; ALFF, Amplitude of low-frequency fluctuation; ReHo, Regional homogeneity; NA, not available.

### Regional differences in GMV

**Figure 2:**
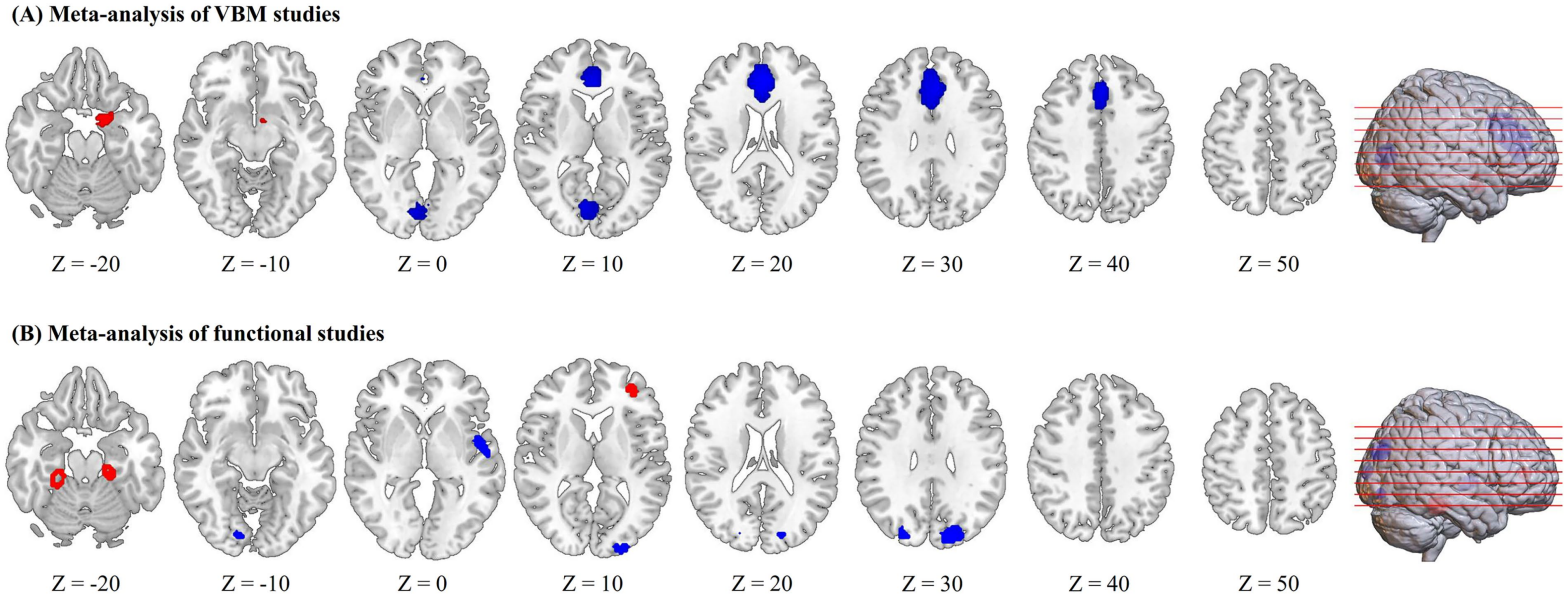
Regions of increased (red) and decreased (blue) gray matter volume or brain activity in HIV-infected patients compared to controls in the meta-analyses of voxel-based morphometry (VBM) studies **(A)** and functional studies **(B)**.

Compared with HIV-negative controls, HIV-infected patients showed higher regional GMV in the left amygdala, as well as lower regional GMV in the bilateral medial PFC/ACC and some visual cortices (bilateral calcarine fissure/surrounding cortex extending to the lingual gyrus and cuneus cortex; Figure 2A; Table 2).

**Table 2:** Meta-analysis results of regional gray matter volume changes in HIV-infected patients compared with controls.

Brain regions	MNI coordinates			SDM z-score	*p*-value	Cluster	
	*x*	*y*	*z*			No. of voxels	Cluster breakdown (no. of voxels)
*HIV+ > HIV-*							
Left amygdala	−20	0	−20	1.038	0.000681221	230	Left parahippocampal gyrus (73); left amygdala (52); left striatum (13); left temporal pole, superior temporal gyrus (14); left olfactory cortex (16); left hippocampus (6)
*HIV+ < HIV-*							
Bilateral medial PFC/ACC	−2	38	20	−2.587	0.000051618	2,111	Left anterior cingulate/paracingulate gyri (647); right anterior cingulate/paracingulate gyri (469); left superior frontal gyrus, medial (372); right superior frontal gyrus, medial (31); left median cingulate/paracingulate gyri (157); right median cingulate/paracingulate gyri (206); left supplementary motor area (18)
Bilateral calcarine fissure/surrounding cortex	10	−78	8	−2.394	0.000113547	506	Left calcarine fissure/surrounding cortex (166); right calcarine fissure/surrounding cortex (118); left lingual gyrus (47); right lingual gyrus (36); left cuneus cortex (7); right cuneus cortex (5)

PFC, Prefrontal cortex; ACC, Anterior cingulate cortex; MNI, Montreal Neurological Institute; SDM, Seed-based *d* Mapping.

### Regional differences in resting-state brain activity

HIV-infected patients exhibited increased regional brain activity in the right fusiform gyrus, left parahippocampal gyrus and left middle frontal gyrus and decreased regional brain activity in the bilateral superior occipital gyrus, left superior temporal gyrus, left middle occipital gyrus and right lingual gyrus (Figure 2B and Table 3).

**Table 3:** Meta-analysis results of regional brain activity changes in HIV-infected patients compared with controls.

Brain regions	MNI coordinates			SDM z-score	*p*-value	Cluster	
	*x*	*y*	*z*			No. of voxels	Cluster breakdown (no. of voxels)
*HIV+ > HIV-*							
Right fusiform gyrus	28	−28	−24	1.207	0.002348185	257	Right fusiform gyrus (104); right cerebellum, hemispheric lobule IV/V (96); right parahippocampal gyrus (42); right cerebellum, hemispheric lobule III (2)
Left parahippocampal gyrus	−18	−28	−22	1.206	0.002373993	186	Left parahippocampal gyrus (72); left fusiform gyrus (32); left cerebellum, hemispheric lobule IV/V (5); left hippocampus (1)
Left middle frontal gyrus	−32	44	10	1.496	0.000418007	98	Left middle frontal gyrus (30); left inferior frontal gyrus (23)
*HIV+ < HIV-*							
Left superior occipital gyrus	−22	−86	26	−1.730	0.000366390	384	Left superior occipital gyrus (153); left cuneus cortex (53); left middle occipital gyrus (33)
Left superior temporal gyrus	−56	−10	0	−1.422	0.003039718	106	Left superior temporal gyrus (58); left rolandic operculum (39); left insula (4); left middle temporal gyrus (3)
Right superior occipital gyrus	22	−82	26	−1.571	0.001279891	94	Right superior occipital gyrus (48)
Left middle occipital gyrus	−26	−96	8	−1.582	0.001181841	88	Left middle occipital gyrus (82)
Right lingual gyrus	16	−82	−10	−1.430	0.002972603	38	Right lingual gyrus (19)

MNI, Montreal Neurological Institute; SDM, Seed-based *d* Mapping.

### Multimodal analysis of structural and functional alterations

Multimodal analysis of structural and functional MRI studies did not find overlapped brain regions with both structural and functional abnormalities in HIV-infected patients.

### Reliability analysis

In the meta-analysis of VBM studies, whole-brain jackknife sensitivity analyses revealed that GMV reductions in the bilateral medial PFC/ACC and bilateral calcarine fissure/surrounding cortex were highly robust, as they were replicable in all study combinations. Increased GMV in the left amygdala was highly replicable, as it remained significant in 10 out of the 11 combinations (Supplementary Table S5). In the meta-analysis of rs-fMRI studies, whole-brain jackknife sensitivity analyses revealed that all clusters showed high replicability and reliability as they remained significant in at least 6 out of the 8 combinations (Supplementary Table S6).

### Heterogeneity and publication bias analyses

In the meta-analysis of VBM studies, there was significant unexplained between-study variability in the bilateral medial PFC/ACC (*p* = 0.000438690, SDM-Z = 3.120) after heterogeneity analyses with *Q* statistics. In the meta-analysis of functional studies, heterogeneity analyses with *Q* statistics showed significant unexplained between-study variability in the left superior temporal gyrus (*p* = 0.000149667, SDM-Z = 2.832). The Egger test was nonsignificant in all clusters in the meta-analysis of VBM studies (Supplementary Table S7), but was significant in the left superior temporal gyrus (*p* = 0.046) in the meta-analysis of functional studies (Supplementary Table S8).

### Meta-regression analysis

The meta-regression analysis revealed that mean age, percentage of males, and mean current CD4 cell count were not linearly associated with either regional GMV or regional brain activity alterations in HIV-infected patients.

## Discussion

The current study provides a unique multimodal view of brain structural and functional alterations in HIV infection. To our knowledge, this is the first whole-brain voxel-wise meta-analysis to evaluate the patterns of change in regional GMV and resting-state brain activity in HIV-infected patients. This multimodal meta-analysis suggested that HIV-infected patients showed structural abnormalities alone in the bilateral medial prefrontal cortex/anterior cingulate cortex, bilateral calcarine cortex and left amygdala, and exhibited functional abnormalities alone in the left middle frontal gyrus, left parahippocampal gyrus, left superior temporal gyrus and visual cortices. No conjoint brain structural and functional abnormalities were observed in HIV-infected patients.

### Structural-specific brain abnormalities in HIV infection

The meta-analysis of structural studies characterized regional GMV reductions in the medial PFC/ACC in HIV-infected patients. The medial PFC/ACC is a core region of the default mode network (35), and contributes to self-relevance, rapid error identification and social functions and mediates the interplay between emotional processes and cognitive functions (36). Similarly, reduced GMV in the ACC has been demonstrated in HIV + individuals compared to HIV- controls using a ROI analytical method, and volumetric abnormality of the ACC was found to be associated with fear recognition impairments in the HIV + group (37). Significant reduced cortical thickness reductions in the prefrontal regions have also been observed in HIV-infected patients compared to HIV-uninfected controls (30). Thus, our findings are consistent with these. Structural abnormalities in the medial PFC/ACC may play important roles in the neurobiology of HIV-related cognitive deficits.

Additionally, our study revealed decreased regional GMV in the calcarine cortex in HIV-infected patients. The calcarine cortex is an important component of the visual network, responsible for visual memory and vision processing (38). Deficits in visual memory and visuospatial ability have been demonstrated in HIV-infected subjects (39, 40). Therefore, structural abnormalities in the calcarine cortex might underline visual-related cognitive dysfunctions in HIV-infected patients.

We also found increased regional GMV in the left amygdala in HIV-infected patients. The etiology of gray matter hypertrophy observed in HIV-infected patients remains unclear since such findings have been rarely reported in individuals with HIV. An increase in GMV could be due to an increase in cell size, neural or glial cell genesis, or spine volume (41, 42). The amygdala has long been associated with emotion and motivation, playing an essential part in processing both fearful and rewarding environmental stimuli (43). Regional GMV alterations in the amygdala are often observed in psychiatric disorders, such as major depressive disorder (44) and anxiety disorder (45). Prior neuroimaging study suggested that HIV infection and high levels of early life stress interacted to increase amygdala volume, which was associated with neurocognitive impairment in HIV-infected patients (46). Another ROI-based structural neuroimaging study also demonstrated increased amygdala volume in HIV + patients compared to controls (37). Thus, our findings are compatible with these previous studies.

### Functional-specific brain abnormalities in HIV infection

Our meta-analysis also demonstrated functional-specific abnormalities in HIV-infected patients. Altered regional brain activity were found in the left middle frontal gyrus, left parahippocampal gyrus, left superior temporal gyrus and visual cortices, without overlapped structural abnormalities.

Specifically, HIV-infected patients showed hyperactivity in the right fusiform gyrus, left parahippocampal gyrus and left middle frontal gyrus. The parahippocampal gyrus is involved in many cognitive functions, including visuospatial processing and episodic memory (47). Converging evidence suggests that the fusiform gyrus plays a pivotal role in high-level visual/cognitive functions, such as facial recognition (48) and recognition of various object features (49). The middle frontal and midcingulate cortices are associated high-order cognitive abilities, such as executive functions and cognitive control (50, 51). The exact mechanism of the hyperactivation of these brain regions in HIV-infected patients remains unclear since little evidence has been reported. One possibility is that HIV-induced neuroinflammation impairs neural efficiency with resultant compensatory increases in neuronal activation. Another possibility is that increased brain activity in these regions perhaps represent physiological responses to HIV infection that protect against the development of cognitive impairment.

Additionally, this meta-analysis revealed hypoactivity in the left superior temporal gyrus in HIV-infected patients. The superior temporal gyrus has been associated with auditory functions and language processing. Evidence from previous studies support our findings. For example, prior study suggested a pattern of hypoperfusion in HIV-infected individuals that involves temporal regions (52). Significant reduced cortical thickness in the lateral temporal regions were also found in HIV-infected individuals (30). Functional deficits in the left superior temporal gyrus might be the neural substrates of impaired language and auditory functions observed in HIV-infected patients. HIV-infected patients also showed decreased brain activity in the occipital regions, including the bilateral superior occipital gyrus, left middle occipital gyrus and right lingual gyrus, compared to HIV-negative controls. The occipital regions are primarily involved in visual processing and low-order cognitive processing. Functional abnormalities of the visual cortices might contribute to impaired visual function in HIV-infected patients. Overall, given the high rates of cognitive disorders in antiretroviral-treated people living with HIV, functional deficits in the temporal and occipital regions may play important roles in the neurobiology of HIV-associated cognitive impairment (2, 53, 54).

Notably, this meta-analysis did not identify overlapped regions with both structural and functional abnormalities in HIV-infected patients. The absence of overlapping regions of structural and functional abnormalities in HIV-infected patients may suggest that these two types of neural changes may occur independently or at different stages of the disease. Neurobiologically, structural damage, such as gray matter loss, often results from long-term neurotoxic effects of HIV-related neuroinflammation and viral proteins, leading to permanent tissue loss. In contrast, functional abnormalities, observed through altered brain activity or connectivity, may reflect early or compensatory responses to neural injury, potentially before structural changes become evident (55). This dissociation emphasizes the complex nature of HIV’s impact on the brain. Clinically, these findings underscore the importance of employing multimodal imaging to capture the full spectrum of neural alterations. Understanding how and when these abnormalities develop can inform targeted interventions, aiming to prevent or mitigate cognitive decline. Future longitudinal studies are necessary to elucidate the progression and relationship between structural and functional changes in HIV-infected patients.

We acknowledge several limitations in this meta-analysis. First, the number of included studies was relatively small, which may affect the power of the statistical analysis. Second, we did not conduct a subgroup meta-analysis to evaluate the effect of antiretroviral therapy on brain structure and function in HIV-infected patients due to a small dataset of untreated patients. Comparative meta-analysis should be conducted to address this issue with more original studies are available in the future; Finally, given the exploratory nature of our analysis, we prioritized detecting potential effects without correction for multiple comparisons, but we acknowledge that this may increase the risk of false positives. Future studies are needed to address this issue.

## Conclusion

This multimodal meta-analysis identified dissociated brain structural and functional alterations in HIV-infected patients. These findings may provide new insights into the neurobiology of HIV-associated neurocognitive impairment. With more and more relevant studies published, future quantitative meta-analysis should explore the potential effects of antiretroviral therapy on brain structure and function in HIV-infected individuals.

## Footnotes

^1^version 5.15, https://www.sdmproject.com/software

^2^ http://sdmproject.com/software/tutorial.pdf
