# Supplementary material for: Divergent structural and functional brain alterations in HIV-infected patients: a multimodal meta-analysis
Source: Front Neurol. 2025 Aug 18;16:1618408. doi: 10.3389/fneur.2025.1618408 (PMC12400857; doi:10.3389/fneur.2025.1618408)
Supplement: Supplementary file 1 [file Table_1.docx]

***Divergent structural and functional brain alterations in HIV-infected patients: a multimodal meta-analysis***

***Supplementary materials***

**Supplementary methods**

***Multimodal analysis***

To examine the overlap of regional GMV and resting-state brain activity alterations, we summarized structural and functional findings in a single meta-analytic map as described elsewhere ^1^. This multimodal meta-analysis aims to ensure that the false-positive rate is not increased compared with that in studies of any single modality. Using a separate meta-analysis, we obtained a probability map of GMV (*p*_GMV_) and resting-state brain activity (*p*_RSBA_) alterations to identify regions with alterations in each modality. The multimodal analysis combined the two probability maps, incorporating the *p* values to identify a union of alterations in both modalities (*U*). The estimation of *U* is straightforward as *U* = *p*_GMV_ + *p*_RSBA_ − *p*_GMV_ × *p*_RSBA_. However, the *U* statistic in its raw form is overly conservative, and to reduce the imbalance between the false-positive and negative rates, *U* was adjusted according to *p* = *U* + (1 − *U*) × 1n (1 − *U*) ^2^. A more stringent probability threshold was employed for this multimodal analysis (*p* < 0.0025) than that used in unimodal meta-analyses.

This multimodal analysis aimed to identify brain regions that showed overlapping structural and functional abnormalities (i.e., conjoint abnormalities); it did not aim to assess correlations between them, either in the same or different brain regions, as it could be that structural deficits in one brain area produce functional alterations in a different area ^2^.

**Supplementary tables**

**Table S1. PRISMA checklist.**

| **Section / topic** | **#** | **Checklist item** | **Reported on page #** |
| --- | --- | --- | --- |
| **TITLE** | | |  |
| Title | 1 | Identify the report as a systematic review, meta-analysis, or both. | Title page |
| **ABSTRACT** | | |  |
| Structured summary | 2 | Provide a structured summary including, as applicable: background; objectives; data sources; study eligibility criteria, participants, and interventions; study appraisal and synthesis methods; results; conclusions and implications of key findings. | Abstract section |
| **INTRODUCTION** | | |  |
| Rationale | 3 | Describe the rationale for the review in the context of what is already known. | Paragraphs 1 to 4 |
| Objectives | 4 | Provide an explicit statement of questions being addressed with reference to participants, interventions, comparisons, outcomes, and study design (PICOS). | Paragraph 5 |
| **METHODS** | | |  |
| Protocol and registration | 5 | Indicate if a review protocol exists, if and where it can be accessed (e.g., Web address), and, if available, provide registration information including registration number. | NA |
| Eligibility criteria | 6 | Specify study characteristics (e.g., PICOS, length of follow-up) and report characteristics (e.g., years considered, language, publication status) used as criteria for eligibility, giving rationale. | Subheading 1 |
| Information sources | 7 | Describe all information sources (e.g., databases with dates of coverage, contact with study authors to identify additional studies) in the search and date last searched. | Subheading 1 |
| Search | 8 | Present full electronic search strategy for at least one database, including any limits used, such that it could be repeated. | Subheading 1 |
| Study selection | 9 | State the process for selecting studies (i.e., screening, eligibility, included in systematic review, and, if applicable, included in the meta-analysis). | Subheading 1 |
| Data collection process | 10 | Describe method of data extraction from reports (e.g., piloted forms, independently, in duplicate) and any processes for obtaining and confirming data from investigators. | Subheading 2 |
| Data items | 11 | List and define all variables for which data were sought (e.g., PICOS, funding sources) and any assumptions and simplifications made. | NA |
| Risk of bias in individual studies | 12 | Describe methods used for assessing risk of bias of individual studies (including specification of whether this was done at the study or outcome level), and how this information is to be used in any data synthesis. | NA |
| Summary measures | 13 | State the principal summary measures (e.g., risk ratio, difference in means). | Subheading 4 |
| Synthesis of results | 14 | Describe the methods of handling data and combining results of studies, if done, including measures of consistency (e.g., I^2^) for each meta-analysis. | Subheading 5 |
| Risk of bias across studies | 15 | Specify any assessment of risk of bias that may affect the cumulative evidence (e.g., publication bias, selective reporting within studies). | Subheading 6 |
| Additional analyses | 16 | Describe methods of additional analyses (e.g., sensitivity or subgroup analyses, meta-regression), if done, indicating which were pre-specified. | Subheading 7 |
| **RESULTS** | | |  |
| Study selection | 17 | Give numbers of studies screened, assessed for eligibility, and included in the review, with reasons for exclusions at each stage, ideally with a flow diagram. | Figure 1 |
| Study characteristics | 18 | For each study, present characteristics for which data were extracted (e.g., study size, PICOS, follow-up period) and provide the citations. | Table 1 |
| Risk of bias within studies | 19 | Present data on risk of bias of each study and, if available, any outcome level assessment (see item 12). | NA |
| Results of individual studies | 20 | For all outcomes considered (benefits or harms), present, for each study: (a) simple summary data for each intervention group (b) effect estimates and confidence intervals, ideally with a forest plot. | Subheadings 2 to 4 |
| Synthesis of results | 21 | Present results of each meta-analysis done, including confidence intervals and measures of consistency. | Subheading 5 |
| Risk of bias across studies | 22 | Present results of any assessment of risk of bias across studies (see Item 15). | Subheading 6 |
| Additional analysis | 23 | Give results of additional analyses, if done (e.g., sensitivity or subgroup analyses, meta-regression [see Item 16]). | Subheading 7 |
| **DISCUSSION** | | |  |
| Summary of evidence | 24 | Summarize the main findings including the strength of evidence for each main outcome; consider their relevance to key groups (e.g., healthcare providers, users, and policy makers). | Discussion section |
| Limitations | 25 | Discuss limitations at study and outcome level (e.g., risk of bias), and at review-level (e.g., incomplete retrieval of identified research, reporting bias). | Limitation section |
| Conclusions | 26 | Provide a general interpretation of the results in the context of other evidence, and implications for future research. | Conclusion section |
| **FUNDING** | | |  |
| Funding | 27 | Describe sources of funding for the systematic review and other support (e.g., supply of data); role of funders for the systematic review. | Funding information |

**Table S2. Quality assessment checklist (when criteria were partially met, 0.5 points were assigned).**

| **Category 1: Participants** | Score (0/0.5/1) |
| --- | --- |
| 1. Patients were evaluated prospectively, specific diagnostic criteria were applied, and demographic data were reported.  2. Comparison participants were evaluated prospectively, with psychiatric and medical illnesses were excluded.  3. Important variables (e.g., age, sex, illness duration, onset, medication status, BMI, HbA1c, intelligence quotient, i.e. IQ, handedness) were checked either by stratification or statistically.  4. Sample size per group > 10. | |
| **Category 2: Methods for image acquisition and analysis** | |
| 5. Whole brain analysis was automated with no a priori regional selection.  6. Coordinates reported in a standard space.  7. The imaging technique used was clearly described so that it could be reproduced.  8. Measurements were clearly described so that they could be reproduced. | |
| **Category 3: Results and conclusions** | |
| 9. Statistical parameters for significant and important non-significant differences were provided.  10. Conclusions were consistent with the results obtained and the limitations were discussed. | |
| TOTAL /10 | |

**Table S3. Technique details for voxel-based morphometry studies included in the meta-analysis.**

| **Datasets** | **Field strength/MRI scanner** | **Head coil** | **Sequence** | **TR/TE/TI (ms)** | **Voxel size (mm^3^)** | **Software** | **FWHM (mm)** | **Covariate** | **Threshold** |
| --- | --- | --- | --- | --- | --- | --- | --- | --- | --- |
| Küper M, et al. (2011) | 1.5 T/Siemens | 8-channel | MPRAGE | 2400/4.38/1200 | 1 × 1 × 1 | SPM5 | 10 | Age and gender | *p* < 0.05 (FDR) |
| Li YF, et al. (2014) | 3.0 T/Siemens | 32-channel | MPRAGE | 1900/2.52/900 | Thickness = 1^*^ | SPM8 | 8 | NA | *p* < 0.001, uncorrected |
| Wilson TW, et al. (2015) | 3.0 T/Philips | NA | 3D-FFE | 8.1/3.7/NA | Thickness = 1^*^ | SPM8 | 8 | NA | *p* < 0.001 (GRF) |
| Wang B, et al. (2016) | 3.0 T/Siemens | NA | SPGR | 1900/2.52/NA | Thickness = 1^*^ | SPM8 | 6 | Age and gender | *p* < 0.05 (FDR) |
| Zhou YW, et al. (2017) | 3.0 T/Siemens | 32-channel | SPGR | 1900/2.52/900 | 1 × 1 × 1 | FSL-VBM | 3 | NA | *p* < 0.05 (FWE) |
| Sanford R, et al. (2017) | 3.0 T/Siemens | 12-channel | MPRAGE | 2400/3.16/1000 | 1 × 1 × 1 | NA | 8 | Age, gender, ethnicity, and education | *p* < 0.05 (FDR) |
| Li JL, et al. (2018) | 3.0 T/Siemens | NA | MPRAGE | 1900/2.1/900 | Thickness = 1^*^ | SPM12 | 8 | Age, gender and TIV | *p* < 0.05 (FWE) |
| Yu X, et al. (2019) | 3.0 T/Siemens | NA | MPRAGE | 5000/2.88/700 | Thickness = 1^*^ | SPM12 | 8 | Age and gender | *p* < 0.05 (FWE) |
| Liu D, et al. (2020) | 3.0 T/Siemens | 32-channel | MPRAGE | 1900/2.52/900 | 1 × 1 × 1 | SPM12 | 8 | Age, gender and TIV | *p* < 0.05 (GRF) |
| Kato T, et al. (2020) | 1.5 T/Philips | 8-channel | 3D-TFE | 8.3/3.8/NA | 1 × 1 × 1 | SPM12 | 8 | Age and TIV | *p* < 0.001, uncorrected |
| Ma Q, et al. (2022) | 3.0 T/Philips | 8-channel | MPRAGE | 1900/3.8/NA | 1 × 1 × 1 | SPM12 | 8 | Age | *p* < 0.05 (FWE) |

**Abbreviations:** MPRAGE = magnetization prepared rapid acquisition gradient-echo; FFE = fast field echo; SPGR = spoiled gradient recall; TFE = turbo field echo; TR/TE/TI = repetition time/echo time/inversion time; FWHM = full-width at half-maximum; TIV = total intracranial volume; FDR = false discovery rate; GRF = Gaussian random field; FWE = family-wise error; NA = not available.

^*^ Details are not available.

**Table S4. Technique details for resting-state brain activity studies included in the meta-analysis.**

| **Datasets** | **Magnetic field strength/MRI scanner** | **Head coil** | **Sequence** | **TR/TE (ms)** | **Voxel size (mm^3^)** | **Software** | **FWHM (mm)** | **Threshold** | **Covariate** | **Measure** |
| --- | --- | --- | --- | --- | --- | --- | --- | --- | --- | --- |
| Wang PY, et al. (2018) | 3.0 T/Siemens | 8-channel | EPI | 2000/30 | Thickness = 4.5^*^ | SPM8, REST | 6 | *p* < 0.05 (AlphaSim) | Age, gender and education | ReHo |
| Yadav SK, et al. (2018) | 3.0 T/GE | 8-channel | EPI | 2500/30 | Thickness = 3^*^ | SPM12 | 6 | *p* < 0.05 (AlphaSim) | Age and gender | ALFF |
| Bak Y, et al. (2018) | 3.0 T/Siemens | NA | EPI | 3000/30 | 3 × 3 × 3 | SPM8, DPARSF | 8 | *p* < 0.05 (AlphaSim) | NA | ALFF |
| Egbert AR, et al. (2018) | 3.0 T/Siemens | 32-channel | EPI | 2000/25 | 3 × 3 × 3 | SPM12 | 6 | *p* < 0.05 (FDR) | Age | ReHo |
| Li RL, et al. (2019) | 3.0 T/Siemens | 32-channel | EPI | 2000/30 | 3.5 × 3.5 × 4.2 | SPM8 | 8 | *p* < 0.05 (AlphaSim) | Age, gender and education | ALFF |
| Sarma MK, et al. (2021) | 3.0 T/Siemens | 16-channel | EPI | 2000/27 | 3.75 × 3.75 × 4 | SPM12, DPABI | 6 | *p* < 0.05 (FWE) | Age and gender | ALFF |
| Ma Q, et al. (2022) | 3.0 T/Philips | 8-channel | EPI | 2000/25 | 1 × 1 × 1 | SPM12, REST | 6 | *p* < 0.05 (FWE) | Age | ALFF |
| Han S, et al. (2022) | 3.0 T/ Siemens | 32-channel | EPI | 2000/30 | 3.5 × 3.5 × 3.5 | DPABI | 4 | *p* < 0.05 (FDR) | NA | ReHo |

**Abbreviations:** EPI = echo-planar imaging; TR/TE = repetition time/echo time; FWHM = full-width at half-maximum; FDR = false discovery rata; FWE = family-wise error; ReHo = regional homogeneity; ALFF = amplitude of low-frequency fluctuation; NA = not available.

^*^ Details are not available.

**Table S5. Reliability (jackknife sensitivity) analyses for brain regions showing gray matter volume alterations in HIV+ vs. HIV-.**

| Discarded study |  | HIV+ > HIV- |  | HIV+ < HIV- | |
| --- | --- | --- | --- | --- | --- |
|  |  | Left amygdala |  | Bilateral MPFC/ACC | Bilateral CAL |
| Küper M, et al. (2011) |  | Y |  | Y | Y |
| Li YF, et al. (2014) |  | Y |  | Y | Y |
| Wilson TW, et al. (2015) |  | Y |  | Y | Y |
| Wang B, et al. (2016) |  | Y |  | Y | Y |
| Zhou YW, et al. (2017) |  | Y |  | Y | Y |
| Sanford R, et al. (2017) |  | Y |  | Y | Y |
| Li JL, et al. (2018) |  | Y |  | Y | Y |
| Yu X, et al. (2019) |  | Y |  | Y | Y |
| Liu D, et al. (2020) |  | Y |  | Y | Y |
| Kato T, et al. (2020) |  | Y |  | Y | Y |
| Ma Q, et al. (2022) |  | N |  | Y | Y |
| Total |  | 10/11 |  | 11/11 | 11/11 |

Abbreviations: MPFC = medial prefrontal cortex; ACC = anterior cingulate cortex; CAL = calcarine fissure and surrounding cortex; Y = yes; N = no.

**Table S6. Reliability (jackknife sensitivity) analyses for brain regions showing brain activity alterations in HIV+ *vs.* HIV-.**

| Discarded study |  | HIV+ > HIV- | | |  | HIV+ < HIV- | | | | |
| --- | --- | --- | --- | --- | --- | --- | --- | --- | --- | --- |
|  |  | R-FFG | L-PHG | L-MFG |  | L-SOG | L-STG | R-SOG | L-MOG | R-LING |
| Wang PY, et al. (2018) |  | Y | Y | Y |  | Y | N | Y | Y | Y |
| Yadav SK, et al. (2018) |  | Y | Y | Y |  | Y | Y | Y | Y | Y |
| Bak Y, et al. (2018) |  | Y | Y | Y |  | Y | N | Y | Y | Y |
| Egbert AR, et al. (2018) |  | Y | Y | Y |  | Y | Y | Y | Y | Y |
| Li RL, et al. (2019) |  | Y | Y | Y |  | Y | Y | N | Y | Y |
| Sarma MK, et al. (2021) |  | Y | Y | Y |  | Y | Y | Y | Y | Y |
| Ma Q, et al. (2022) |  | N | N | Y |  | Y | Y | Y | Y | Y |
| Han S, et al. (2022) |  | Y | Y | N |  | N | Y | N | N | N |
| Total |  | 7/8 | 7/8 | 7/8 |  | 7/8 | 6/8 | 6/8 | 7/8 | 7/8 |

**Abbreviations:** FFG = fusiform gyrus; PHG = parahippocampal gyrus; MFG = middle frontal gyrus; SOG = superior occipital gyrus; STG = superior temporal gyrus; MOG = middle occipital gyrus; LING = lingual gyrus; R = right; L = left; B = bilateral; Y = yes; N = no.

**Table S7. Results of funnel plots and Egger test for brain regions showing gray matter volume alterations in HIV+ *vs.* HIV-.**

| Brain areas | MNI coordinates  (x, y, z) | Funnel plots | Egger test |
| --- | --- | --- | --- |
|  | x, y, z |  |  |
| Left amygdala | -20, 0, -20 | **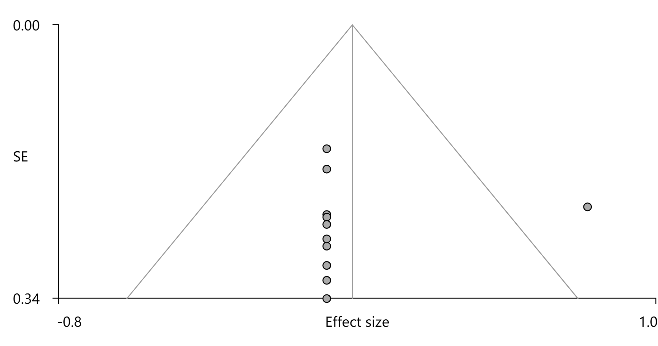** | *t* = -0.01, *p* = 0.989 |
| Bilateral medial PFC/ACC | -2, 38, 20 | **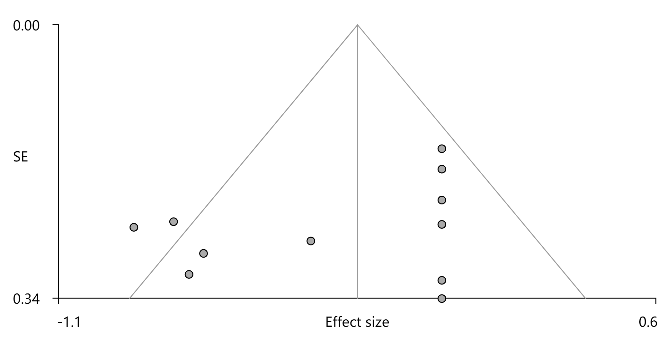** | *t* = -1.58, *p* = 0.149 |
| Bilateral calcarine fissure/surrounding cortex | 10, -78, 8 | **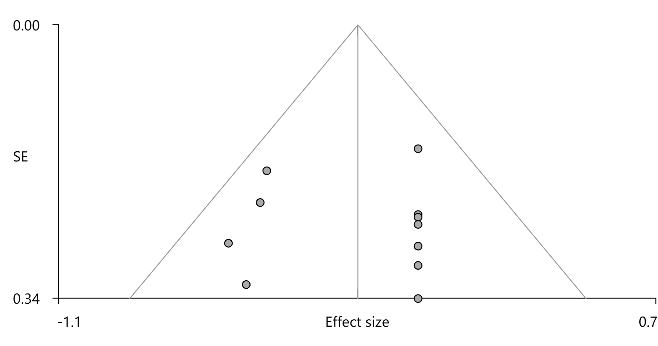** | *t* = -0.13, *p* = 0.896 |

**Abbreviation:** PFC = prefrontal cortex; ACC = anterior cingulate cortex; MNI = Montreal Neurological Institute

**Table S8. Funnel plots and Egger test results for brain regions showing resting-state brain activity alterations in HIV+ *vs.* HIV-.**

| Brain areas | MNI coordinates  (x, y, z) | Funnel plots | Egger test |
| --- | --- | --- | --- |
|  | x, y, z |  |  |
| Right fusiform gyrus | 28, -28, -24 | **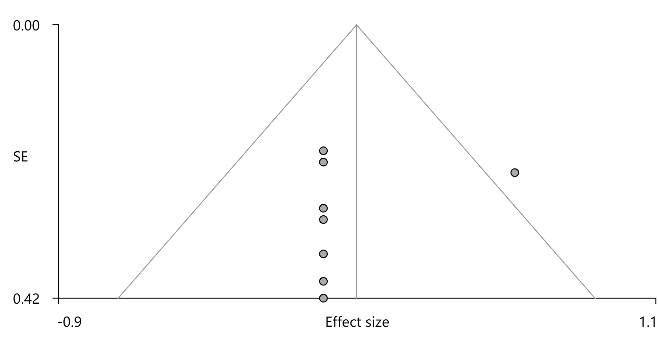** | *t* = -0.50, *p* = 0.636 |
| Left parahippocampal gyrus | -18, -28, -22 | **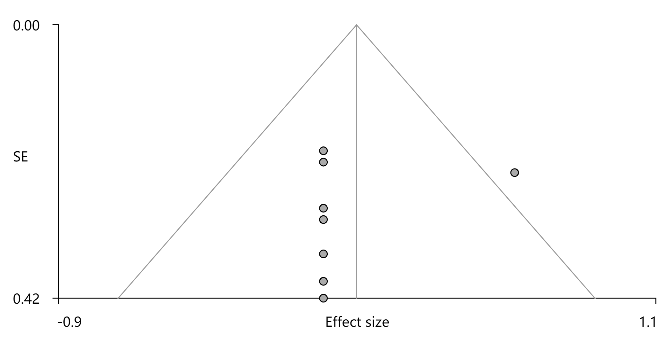** | *t* = -0.50, *p* = 0.636 |
| Left middle frontal gyrus | -32, 44, 10 | **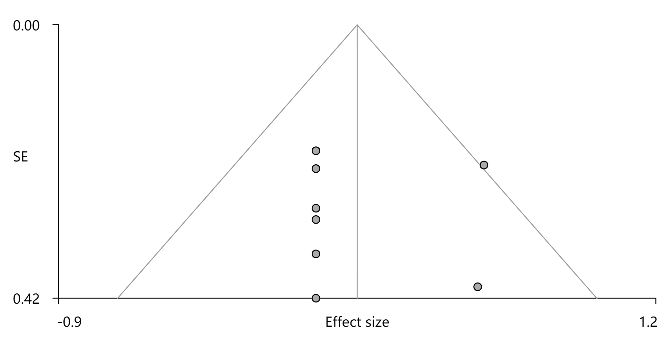** | *t* = -0.03, *p* = 0.977 |
| Left superior occipital gyrus | -22, -86, 26 | **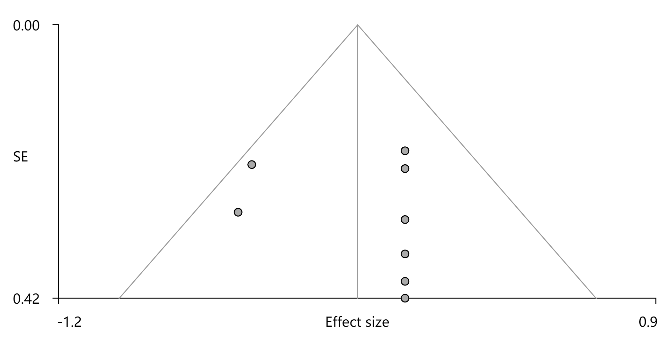** | *t* = 0.35, *p* = 0.736 |
| Left superior temporal gyrus | -56, -10, 0 | **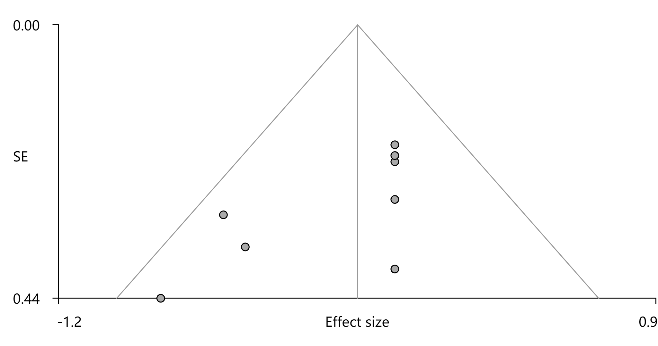** | ***t* = -2.51, *p* = 0.046** |
| Right superior occipital gyrus | 22, -82, 26 | **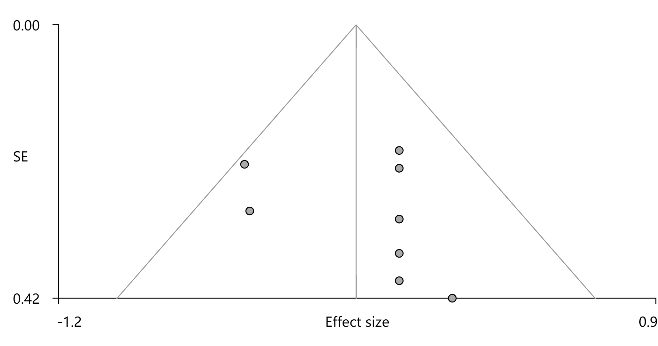** | *t* = 0.62, *p* = 0.561 |
| Left middle occipital gyrus | -26, -96, 8 | **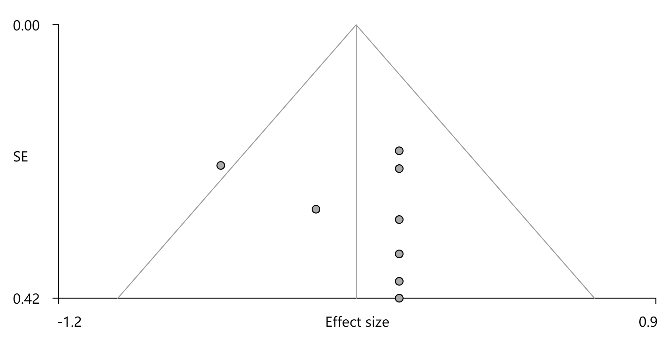** | *t* = 0.60, *p* = 0.568 |
| Right lingual gyrus | 16, -82, -10 | **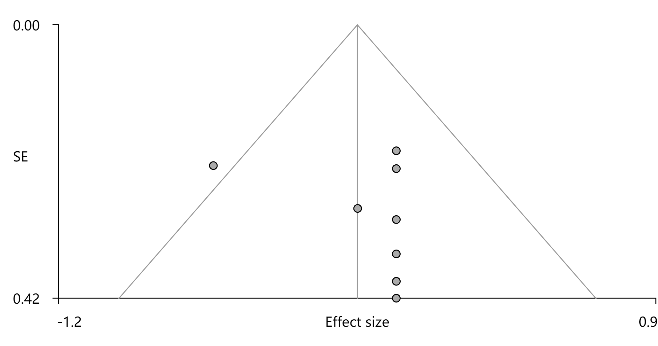** | *t* = 0.69, *p* = 0.517 |

**Abbreviation:** MNI = Montreal Neurological Institute

**References**

1. Radua J, Romeo M, Mataix-Cols D, Fusar-Poli P. A general approach for combining voxel-based meta-analyses conducted in different neuroimaging modalities. Current Medicinal Chemistry 2013;20:462-466.

2. Radua J, Borgwardt S, Crescini A, et al. Multimodal meta-analysis of structural and functional brain changes in first episode psychosis and the effects of antipsychotic medication. Neuroscience and Biobehavioral Reviews 2012;36:2325-2333.
